# Comparative study of wound infection between elective and emergency abdominal surgeries: a retrospective cohort study

**DOI:** 10.1097/MS9.0000000000000549

**Published:** 2023-04-07

**Authors:** Sarosh Khan Jadoon, Raja Muhammad Ijaz Khan, Taufeeq Ahmed Khan, Naheed Akhtar, Yasir Qayyum, Kamlesh Kumar, Rahul Robaish Kumar, Aarti, Ramsha Shahab, Muhammad Sohaib Asghar

**Affiliations:** a Department of General Surgery, SKBZ CMH Muzaffarabad, Azad Jammu and Kashmir; b Department of Internal Medicine, Jinnah Sindh Medical University; c Department of Internal Medicine, Jinnah Postgraduate Medical Center; d Department of Internal Medicine, Dow University of Health Sciences – Ojha Campus, Karachi, Pakistan

**Keywords:** abdominal surgery, elective, emergency, surgical site infection

## Abstract

The objective of this study was to determine the frequency of wound infection among patients with abdominal surgeries and to compare the surgical site infection following elective versus emergency abdominal surgeries in a tertiary care hospital.

**Subjects and methods:**

All patients who fulfilled the inclusion criteria in the Department of General Surgery were included in the study. After taking informed written consent history was taken, clinical examination and patients were divided into two groups: group A (elective abdominal surgery) and group B (emergency abdominal surgery), patients in both groups were compared for the outcome that is surgical site infection.

**Result:**

A total of 140 patients who underwent abdominal surgery were included. Wound infection in abdominal surgeries was noted in 26 patients (18.6%), in group A wound infection was noted in 7 (5%), while in group B wound infection was seen in 19 (13.6%).

**Conclusion:**

The rate of wound infection in patients with abdominal surgeries was not low among the study population and the rate of wound infection was higher in emergency abdominal surgeries as compared with elective abdominal surgeries.

## Introduction

Highlights

- This study was conducted to determine the frequency of wound infection among patients with abdominal surgeries.
- We compared the surgical site infection (SSI) following elective versus emergency abdominal surgeries and found higher rates in emergent care.
- There was no significant difference of wound infection was noted in age, sex, BMI, and place of residence between the two groups.

According to estimates, eight million people worldwide suffer from wounds, whether they are infected or not^1^. In the USA, 2% of the entire population is affected by chronic wounds^2^, and similar data have been reported in European countries^3^. Chronic wounds are more common as people age, and diabetic and obese patients are more likely to acquire them due to a number of factors, including hyperglycemia, impaired vascular status, and neuropathy.

Chronic wound infections could impede recovery, which would have negative clinical effects (increased pain, lower quality of life), as well as place a heavy financial burden on healthcare systems. Chronic wound infection care is difficult and necessitates a multidisciplinary strategy. It may also be difficult to distinguish between an infected wound and a chronic, noninfected wound. In fact, nontraditional signs may characterize chronic wound infections, including increased pain, friable granulation tissue, delayed wound healing beyond expectation, wound breakdown, and foul odor that may be not easy identified by nonexpert personnel^4^. To reduce the danger of infection and undesirable results, inappropriate management should be avoided when treating chronic wounds.

Surgical wound infection is a common postoperative complication and causes significant postoperative morbidity and mortality, prolongs hospital stay, and adds between 10 and 20% to hospital costs. Even if it is impossible to completely eradicate wound infection, bringing the infection incidence down to a low level could have a positive impact on patient comfort and the amount of medical resources required^5^. Any purulent discharge from a closed surgical incision, together with signs of inflammation of the surrounding tissue should be considered as wound infection, irrespective of whether microorganisms can be cultured. Within 30 days of surgery, infection at the incision site is possible; however, wounds that have closed and largely healed are not regarded as infected.

Wounds that have a slight quantity of clear discharge fall into the intermediate categories of wounds that may or may not be infected. These wounds may be considered as ‘possibly’ or ‘probably’ infected. To encompass infections of organs or spaces deep below the skin and soft tissues, such as the peritoneum and bone, the Surgical Wound Infection Task Force changed the term ‘surgical wound infection’ to ‘surgical site infection’ in 1992. SSI is further classified into superficial site infection and organ or space infection^6^.

According to microbiologists, healthy intact skin’s primary function is to regulate microbial populations that exist on the skin’s surface and prevent underlying tissue from becoming colonized and invaded by potential pathogens. A wound that exposes subcutaneous tissue creates a moist, warm, and nutrient-rich environment that is ideal for the colonization and growth of microorganisms. However, factors like wound type, depth, location, and quality as well as the amount of tissue perfusion will affect the quantity and variety of microorganisms in any wound and the antimicrobial efficacy of the host immune response. A traumatic wound is likely to have foreign material and devitalized tissue, whereas the microflora associated with clean, surgical wounds would be anticipated to be low. So the former is likely to facilitate microbial proliferation unless early prophylactic antibiotic treatment and surgical debridement is implemented^7^.

Since wound colonization is most frequently polymicrobial^8^, involving numerous microorganisms that are potentially pathogenic, any wound is at some risk of becoming infected^9^. In the event of infection, a wound fail to heal, the patient suffers increased trauma, treatment costs rise, and general wound management practices become more resource-demanding^10^. Following head and neck surgery, a review of postsurgical wound infections showed an increase in the average inpatient time from 14 days when wounds healed without complications to 24 days when the wounds became infected^11^. Zoutman *et al*.^12^ in their analysis of 108 postsurgical wounds concluded that 10.2 days per case was directly attributable to wound infection and that the associated hospital cost was $3937 per infected patient.

As a result, there is reason for concern among medical professionals regarding the risk of wound infection, not only due to the increased trauma to the patient but also due to the burden it puts on financial resources and the growing demand for cost-effective management within the healthcare system. Clinically, since the 1960s, the usage of occlusive dressings has increased in tandem with concerns about wound infection. To maintain a moist and ideal environment for wound healing, dressings like hydrocolloids, polyurethane films, and polyurethane foams are used. Although they have been reported to encourage microbial proliferation in wounds^13^ the infection rate is lower under occlusive dressings than under conventional dry dressings^14^ and wound healing is not impaired.

Although microorganisms are responsible for wound infection, widespread controversy still exists regarding the exact mechanisms by which they cause infection and also their significance in nonhealing wounds that do not exhibit clinical signs of infection. One school of thought is that the density of microorganisms is the critical factor in determining whether a wound is likely to heal^15^. However, a second school of thought argues that the presence of specific pathogens is of primary importance in delayed healing^16^, while yet others have reported microorganisms to be of minimal importance in delayed healing^17^,^18^.

The general consensus is that the burden of SSIs is higher in emergency surgeries when compared with elective surgeries. However, very few previously published studies are supporting or disproving this consensus. Furthermore, we came across very rare local studies whose main focus was to compare the frequency of SSI between elective and emergency surgeries. So, this study has been conducted with the aim to compare the SSI following elective versus emergency abdominal surgeries in our local population. The study results would help to identify the type of surgery which involved more frequent wound infections. On the basis of these results, preventive and corrective measure would be devised to reduce the overall morbidity associated with that type of surgery in our local population.

## Materials and methods

Study design: Comparative retrospective cohort.

Study setting: Department of General Surgery, Sheikh Khalifa bin Zayed/Combined Military Hospital, Muzaffarabad.

Duration: Six months from 1 September 2021 to 28 February 2022.

Sample size: Sample size was calculated by using the WHO sample size calculator keeping the following parameters:

Significance level: 95%.

Anticipated population: 10%^15^

Absolute precision: 5%

*N*=140.

Proportional sampling was done and 70 patients in each group were taken.

Sampling technique: nonprobability consecutive sampling.

Sample selection:

Inclusion criteria:

- Patient admitted for abdominal surgery either elective or emergency.
- Any sex with age from 15 to 70 years.
- Patients having no comorbidity like diabetes mellitus, hypertension, and anemia.
  Exclusion criteria:
- Patients with preexisting skin infections as confirmed through physical examination.
- Patients with previous abdominal surgery.
- Patients who are not fit for general anesthesia (i.e. American Society of Anesthesiologists grade >2 Annexure-II).
- Patients who underwent gynecologic or laparoscopic surgery.
- Patients who received antibiotics for more than 1 week before surgery as confirmed through clinical history.
- Patients who are receiving corticosteroids as confirmed through clinical history.

### Data collection procedure

Permission and approval for conducting the study was sought from the hospital research ethics committee (DME/437/2020). Patients who were candidates for abdominal surgery either emergency or elective and fulfilling the inclusion criteria were enrolled for the study from the Surgery Department of Sheikh Khalifa bin Zayed/Combined Military Hospital, Muzaffarabad. A detailed clinical history of each patient was obtained starting with history of presenting complaint and a thorough general physical examination was done by the trainee researcher himself. All routine laboratory tests necessary before abdominal surgery were performed. Patients were divided into two groups A and B on the basis of type of surgery performed. In group A, all the patients with elective abdominal surgery were added, while in group B, patients who undergo emergency abdominal surgery were added. Shaving of the operative site right before surgery with a clipper was done.

Patients were kept nil by mouth at least 8 h before abdominal surgery and given an injection ceftriaxone 1 g and injection metronidazole 500 mg intravenous 1 h before the incision. Routine aseptic precautions were taken like using autoclaved gowns, drapes, sterile glove, and instruments.

Standard surgical scrub followed by the protocol adopted by our hospital was followed before performing the operation. All cases were operated under general or spinal anesthesia. Abdominal surgery was performed by the experienced consultant surgeon as per standard protocol. Patients were kept nil by mouth for 24 h, oral liquids were started after 24 h. Standard postoperative medications were given to both groups. Drain was removed after 48–72 h if drain contents were less than 10 ml. Patients were kept in the hospital for 3 days and would be followed up for 1 month on weekly basis. Wound infection was diagnosed as per defined in operational definition. All the demographic details and study results would be recorded by the principal researcher on the proforma. STROCCS guidelines were followed to report the findings of the study^19^.

### Data analysis procedure

Data was entered and analyzed using SPSS, version 20. Mean±SD was presented for quantitative variables like age, BMI, height, and weight. Frequency and percentages was calculated for qualitative variables like sex, place of residence, surgical procedure performed (elective/emergency surgery), hypertension, and frequency of wound infection. Both the groups were compared for incidence of wound infection. The chi-square test would be applied and a *P*-value less than 0.05 was considered statistically significant. Effect modifiers like age, sex, BMI, and place of residence were controlled by stratification in both groups. Poststratification chi-square test was applied and a *P*-value less than 0.05 was considered statistically significant.

## RESULTS

A total 140 patients who underwent abdominal surgery were selected to conduct this study. Patients were divided into two groups: group A patients were with elective abdominal surgery and group B patients were with emergency abdominal surgery. Group ‘A’ included 70 subjects of which 44 (31.4%) were male, while 26 (18.6%) were female, with mean age of 40.785±12.335 years, while group ‘B’ also included 70 patients of which 47 (33.6%) were male, while 23 (16.4%) were female, with mean age 41.057±11.379 years (as shown in Tables 1 and 2).

**Table 1:** Age distribution with respect to groups

	*n* (%)		
Age groups (years)	Group A	Group B	*P*
18–42	34 (24.3)	37 (26.4)	0.912
43–65	36 (25.7)	33 (23.6)	
Total	70 (50)	70 (50)	
Mean±SD	40.785±12.335	41.057±11.379	

**Table 2:** Sex distribution with respect to groups (*N*=154)

	*n* (%)			
Sex	Group A	Group B	Total	*P*
Male	44 (31.4)	47 (33.6)	91 (65)	0.443
Female	26 (18.6)	23 (16.4)	49 (35)	
Total	70 (50)	70 (50)	140 (100)	

In group ‘A’, the mean height was 165.82±10.12 cm, while in group ‘B’, the mean height was 161.785±12.973 cm (as shown in Table 3). In group ‘A’, the mean weight was 73.80±11.90 kg, while in group ‘B’, the mean weight was 73.10±13.88 kg (as shown in Table 4). In group ‘A’, the mean BMI was 26.13±2.31 kg/m^2^, while in group ‘B’, the mean BMI was 26.184±2.670 kg/m^2^ (as shown in Table 5).

**Table 3:** Height distribution with respect to groups

	*n* (%)		
Height groups (cm)	Group A	Group B	*P*
97–143	2 (1.4)	4 (2.9)	0.414
144–188	68 (48.6)	66 (47.1)	
Total	70 (50)	70 (50)	
Mean±SD	165.828±10.122	161.785±12.973	

**Table 4:** Weight distribution with respect to groups

	*n* (%)		
Weight groups	Group A	Group B	*P*
49-82 kg	55 (39.3)	51 (36.4)	0.495
83-116 kg	15 (10.7)	19 (13.6)	
Total	70 (50)	70 (50)	
Mean±SD	73.800±11.903	73.100±13.886	

**Table 5:** BMI distribution with respect to groups

	*n* (%)		
BMI groups (kg/m^2^)	Group A	Group B	*P*
18–26.5	40 (28.6)	41 (29.3)	0.810
26.6–34	30 (21.4)	29 (20.7)	
Total	70 (50)	70 (50)	
Mean±SD	26.132±2.318	26.184±2.670	

In group A, place of residence was urban in 44 (31.4%) and rural in 26 (18.6%) patients, while in group B, place of residence was urban in 46 (32.9%) and rural in 24 (17.1%) patients, as shown in Table 6. Wound infection in abdominal surgeries was noted in 26 patients (18.6%), as shown in Table 7. In group A, wound infection was noted in 7 (5%), while in group B, wound infection was seen in 19 (13.6%), as shown in Table 8. The frequencies of age groups, sex, BMI, and place of residence groups were calculated according to wound infection. The results are presented in Tables 9, 10, 11, and 12, respectively.

**Table 6:** Place of residence distribution with respect to groups (*N*=140)

	*n* (%)			
Place of residence	Group A	Group B	Total	*P*
Urban	44 (31.4)	46 (32.9)	90 (64.3)	0.634
Rural	26 (18.6)	24 (17.1)	50 (35.7)	
Total	70 (50)	70 (50)	140 (100)	

**Table 7:** Frequency distribution of wound infection in abdominal surgeries (*N*=140)

Wound infection	*n* (%)
Yes	26 (18.6)
No	114 (81.4)
Total	140 (100)

**Table 8:** Wound infection distribution with respect to groups (*N*=140)

	*n* (%)			
Wound infection	Group A	Group B	Total	*P*
Positive	7 (5)	19 (13.6)	26 (18.6)	0.420
Negative	63 (45)	51 (36.4)	114 (81.4)	
Total	70 (50)	70 (50)	140 (100)	

**Table 9:** Wound infection between two groups with respect to age (*N*=140)

		Wound infection [*n* (%)]		
Age groups (years)	Groups	Positive	Negative	*P*
18–42	A	4 (2.8)	30 (21)	0.632
	B	10 (7)	27 (18.9)	
43–65	A	3 (2.1)	33 (23.1)	0.982
	B	9 (6.3)	24 (16.8)	

**Table 10:** Wound infection between two groups with respect to sex (*N*=140)

		Wound infection [*n* (%)]		
Sex groups	Groups	Positive	Negative	*P*
Male	A	5 (3.5)	39 (27.3)	0.621
	B	14 (9.8)	33 (23.1)	
Female	A	2 (1.4)	24 (16.8)	0.477
	B	5 (3.5)	18 (12.6)	

**Table 11:** Wound infection between two groups with respect to BMI (*N*=140)

		Wound infection [*n* (%)]		
BMI groups (kg/m^2^)	Groups	Positive	Negative	*P*
18–26.5	A	5 (3.5)	35 (24.5)	0.421
	B	13 (9)	28 (19.6)	

**Table 12:** Wound infection between two groups with respect to place of residence (*N*=140)

		Wound infection [*n* (%)]		
Place of residence groups	Groups	Positive	Negative	*P*
Urban	A	6 (4.2)	38 (27)	0.187
	B	10 (7)	36 (25)	
Rural	A	1 (0.69)	25 (17.5)	0.159
	B	9 (6.3)	15 (10.5)	

In our study, there was no significant difference in wound infection was noted in age, sex, BMI, and place of residence between the two groups.

## Discussion

In our study, the wound infection in abdominal surgeries was noted in 26 patients (18.6%) and in group A, wound infection was noted in 7 (5%), while in group B, wound infection was seen in 19 (13.6%) as compared with Janugade *et al.* 20, in their study, reported that the incidence of infection among emergency surgery was 26.3% and among elective is 6.5%. In another study, Dhaigude *et al*.^21^ compared the incidence of postoperative wound complications following emergency and elective abdominal surgery and they found that 10% of patients of emergency abdominal surgery, while 12% of patient with elective abdominal surgery suffered with postoperative wound complications. In Tan *et al*.’s^22^ study, the incidence of SSIs in elective surgery is higher at 19.4%, while its incidence in emergency surgery is 15.47%. However, this finding is not statistically significant (odds ratio=1.315, 95% CI: 0.636–2.722). The higher statistic in elective cases can be attributed to the longer operative duration associated with major surgeries. The majority of studies comparing elective and emergency general procedures has come to the conclusion that the incidence is higher in emergency surgery, which is in contradiction to the findings presented here^23^. Only a handful demonstrated a higher incidence in elective surgeries.

In literature, the correlation between surgeon seniority and SSI outcome in general surgery is an infrequently explored field. However, studies available, such as those carried out by Bandaru and Ahmed link a correlation between junior surgeons and SSI incidence^24^.

There was no difference in the incidence of SSI between the sexes. Literature is currently mixed, with no general consensus^25^. Multivariable analysis identified open surgical approach, emergency operation, length of the operation, and male sex as independent predictors of SSI. Open surgical approach and emergency surgery were documented as risk factors for SSI in previous reports^26^. Although there is no consensus regarding why male patients are predisposed to SSI, studies have shown that, in laparoscopic cholecystectomy, male sex is a predictor of longer and more difficult operations and has a higher rate of conversion^27^. In addition, it is known that there are sex differences in skin colonization that may be associated with differences in skin thickness, sebum production, and skin pH^28^.

In addition, an association was found between SSI and ethnically Chinese patients. However, other studies revealed no difference in incidence between ethnic groups^29^. Comorbidities in this study were not found to contribute to SSI risk. However, studies such as those by Everhart *et al*.^30^ and Khan *et al*.^31^ show a close association between the incidence of infection and the presence of comorbidities^32^,^33^. Khan *et al*.^31^ further illustrated the potential use of the American Society of Anesthesiologists score and the Charlson Comorbidity Index to quantify comorbidity severity and identify SSI risk.

Postoperative drain usage was significantly associated with an increase in SSI incidence. This is in agreement to multiple studies describing the relationship between prolonged drain use (>24 h) and increased SSI risk. Tang *et al*.^34^ attributed it to how drain use was more prevalent in more complicated surgeries and its effect as a foreign body.

In our study ceftriaxone was used as a prophylaxis antibiotic, because the role of prophylactic antibiotics to prevent SSI have found to be overwhelming evidence pointing to its benefit. However, a continuation of use postsurgery provides no additional protection.^35^ A recent systematic review by de Jonge *at al*.^36^ concluded that timing of administration made a significant difference in SSI incidence, with the ideal time being less than 120 min before incision.

The limitations may include a single-center evaluation with no randomization of study participants. The sample size used was also based on a convenience sampling method hence may lead to bias and cannot contribute to the generalizability of the findings. There may be some biases for heterogeneity of incisions such as midline, Kocher, Mcburney, lower midline, etc. laparotomies, time of surgeries, which organ underwent surgery, using external materials into the abdominal cavity like meshes, using drainage tubes and types of them, the number of people present in the operating room, type of anesthesia, etc. Thus, this is not a completely controlled study.

## Conclusion

The rate of wound infection among patients with abdominal surgeries was not found low and the rate was higher in emergency abdominal surgeries as compared with elective abdominal surgeries.

## Ethical approval

Ethical review was obtained from the institutional review board of CMH Muzaffarabad (Ref:App.#D.M.E/2020/437) issued on 3 November 2020.

## Consent

Written informed consent was not required from the study participants before inclusion in the study due to the retrospective nature of data collection.

## Sources of funding

None to declare.

## Authors' contribution

S.K.J. and R.M.I.K. conceived the idea. T.A.K., S.K.J., Y.Q., and R.R.K. collected the data. R.M.I.K, N.A., and R.S. analyzed and interpreted the data. N.A., T.A.K., and M.S.A. did write up the manuscript. R.S., S.K.J., M.S.A., and T.A.K. reviewed and revised the manuscript for intellectual content critically. All authors approved the final version of the manuscript.

## Conflicts of interest disclosure

The authors declare that they have no financial conflict of interest with regard to the content of this report.

## Research registration unique identifying number (UIN)

1. Name of the registry: CMH Muzaffarabad
2. Unique identifying number or registration ID: (UIN: D.M.E/2020/437).
3. Hyperlink to your specific registration (must be publicly accessible and will be checked):

## Guarantor

Muhammad Sohaib Asghar.

## Provenance and peer review

Externally peer reviewed, not commissioned.
